# Disease Impact and Burden of Psoriasis Vulgaris in Visible Body Areas in Japan: A Qualitative Study

**DOI:** 10.1111/1346-8138.17835

**Published:** 2025-07-16

**Authors:** Koki Endo, Yuko Higaki, Ami Cho, Yoshiyuki Sugimoto, Kyoko Ikumi, Miyu Okamura, Moe Matsuo, Yayoi Tada

**Affiliations:** ^1^ The Jikei University Kashiwa Hospital Chiba Japan; ^2^ Wakamatsu‐Cho Mental and Skin Clinic Tokyo Japan; ^3^ AbbVie GK Tokyo Japan; ^4^ Syneos Health Tokyo Japan; ^5^ Teikyo University Tokyo Japan

**Keywords:** patient‐reported outcomes, psoriasis vulgaris, qualitative research, quality of life, social behavior

## Abstract

International studies describe considerable multi‐faceted burdens for patients living with psoriasis vulgaris. However, there is few research on the experience in the life of patients with psoriasis vulgaris in Japan, particularly regarding psychosocial impacts when symptoms are visible parts of their body. A qualitative descriptive study using semi‐structured one‐on‐one interviews was conducted with 23 patients. Interviews focused on the impact of psoriasis vulgaris on motivation, daily life, social interactions, life course decisions, and psychological burdens, and the transcripts were distilled into discrete qualitative concepts. Common concepts elicited from patients included changes in clothing preferences and reluctance to engage in public activities like visiting public baths and pools due to their visible skin lesions. Many patients reported encountering misconceptions about their condition, which impacted their social relationships. Notably, effective treatment led to positive life changes. The adoption of a more patient‐centered treatment design may reduce the unique psychological challenges faced by individuals living with visible psoriasis vulgaris symptoms and increase their quality of life.

## 1 Introduction

Psoriasis is a common inflammatory skin disease characterized by red, scaly, and well‐demarcated skin lesions formed by the hyperproliferation of epidermal keratinocytes [1]. The location of the lesions influences the burden on patients, from self‐esteem to financial challenges caused by reduced productivity at work [2, 3, 4]. Additionally, disease management may reduce quality of life (QoL). For example, topical agents can be time‐consuming or may stain clothing/skin [4].

“Visible skin diseases” is widely used to differentiate hand and face skin conditions. Richard et al. [5] used the Dermatology Life Quality Index (DLQI) to study QoL in patients with eight skin diseases, including psoriasis vulgaris (PsO), across multiple countries. Their findings revealed that patients with visible lesions in exposed areas suffer more than those with less visible lesions.

There are some examples of studies on the burden of PsO in Japan [6, 7]. However, the cumulative effects of the economic and social impact of PsO, such as the impact on career, social relationships, and life course decisions, have not been fully explored [8, 9]. Also, the impact of PsO‐related skin lesions localized to visible areas has received limited attention in QoL research in Japan.

Detailed insight into the disease burden could highlight the need for early intervention and patient‐centric treatment strategies for PsO patients in Japan. This study aims to uncover key concepts of the psychosocial burden through qualitative methods, focusing on the unique challenges faced by patients with visible PsO.

## 2 Methods

A qualitative descriptive study was conducted using 1‐h semi‐structured interviews by two researchers (MO and MM) trained in patient‐reported outcomes. Participants chose a preferred interview method from web, in‐person, and telephone. All interviews were audio‐recorded and transcribed. Enrollment ID numbers ensured anonymity.

The interview guide, developed specifically for this study, included questions derived from previous PsO studies. Questions addressed psychosocial burdens such as impacts on daily living, relationships, and life course decisions (Table S1). A pilot test was conducted with three initial patients to assess the quality of the interview methodology and the guide.

Patient recruitment was carried out by an agency using a physician database. Twenty eligible patients provided informed consent and were assured that their participation was voluntary and would not affect their current/future treatments. The study enrolled adults aged 18 and older with mild‐to‐severe PsO involving visible areas (e.g., face, hands) receiving treatment.

Data were analyzed qualitatively using Atlas.ti software. Two researchers (MO and MM) coded interview transcripts using a codebook developed during pilot testing to ensure consistency. Concepts were grouped into sub‐categories and further clustered into larger categories based on their similarities to address the research questions. Demographics and baseline characteristics were summarized descriptively.

## 3 Results

Twenty‐three patients with PsO (21 recruited through hospitals or clinics, two via a patient advocacy group) participated in interviews. Their demographic and clinical characteristics are shown in Table 1.

**TABLE 1:** Summary of demographic and clinical characteristics of patients (*N* = 23).

Characteristics	*N* (%) or mean (SD)
*Gender*	
Male	18 (78.3)
*Age*	
Mean	51.9 (13.0)
20–29 years old	1 (4.3)
30–39 years old	3 (5.9)
40–49 years old	7 (30.4)
50–59 years old	5 (21.7)
60 years old and more	7 (30.4)
*Educational level*	
University or graduate school	10 (43.5)
Junior college or technical college	3 (13.0)
High school	8 (34.8)
Junior high school	2 (8.7)
*Employment status*	
Employed (Full time)	17 (73.9)
Employed (Part time)	3 (13.0)
Unemployed	1 (4.3)
Retired	2 (8.7)
*Marital status*	
Married	14 (60.9)
Single (with marital history)	5 (21.7)
Single (without marital history)	4 (17.4)
*Current severity of PsO* ^a^	
Mild	3 (13.0)
Moderate	12 (52.2)
Severe	8 (34.8)
*Time since diagnosis*	
< 1 year	2 (8.7)
1–2 years	2 (8.7)
3–5 years	3 (13.0)
6–10 years	1 (4.3)
11–20 years	8 (34.8)
More than 20 years	7 (30.4)
*Current PsO treatment group (any)*	
Any ointment	21 (91.3)
Any oral medicine	8 (34.8)
Any phototherapy	3 (13.0)
Any biologics	7 (30.4)
*Whether “recovery of skin lesions in visible areas” was/is included in patient's treatment goals*	
Yes	17 (73.9)

Abbreviations: PsO, psoriasis vulgaris; SD, stable disease.

^a^ Assessed by physicians at the time of recruitment.

**FIGURE 1:**
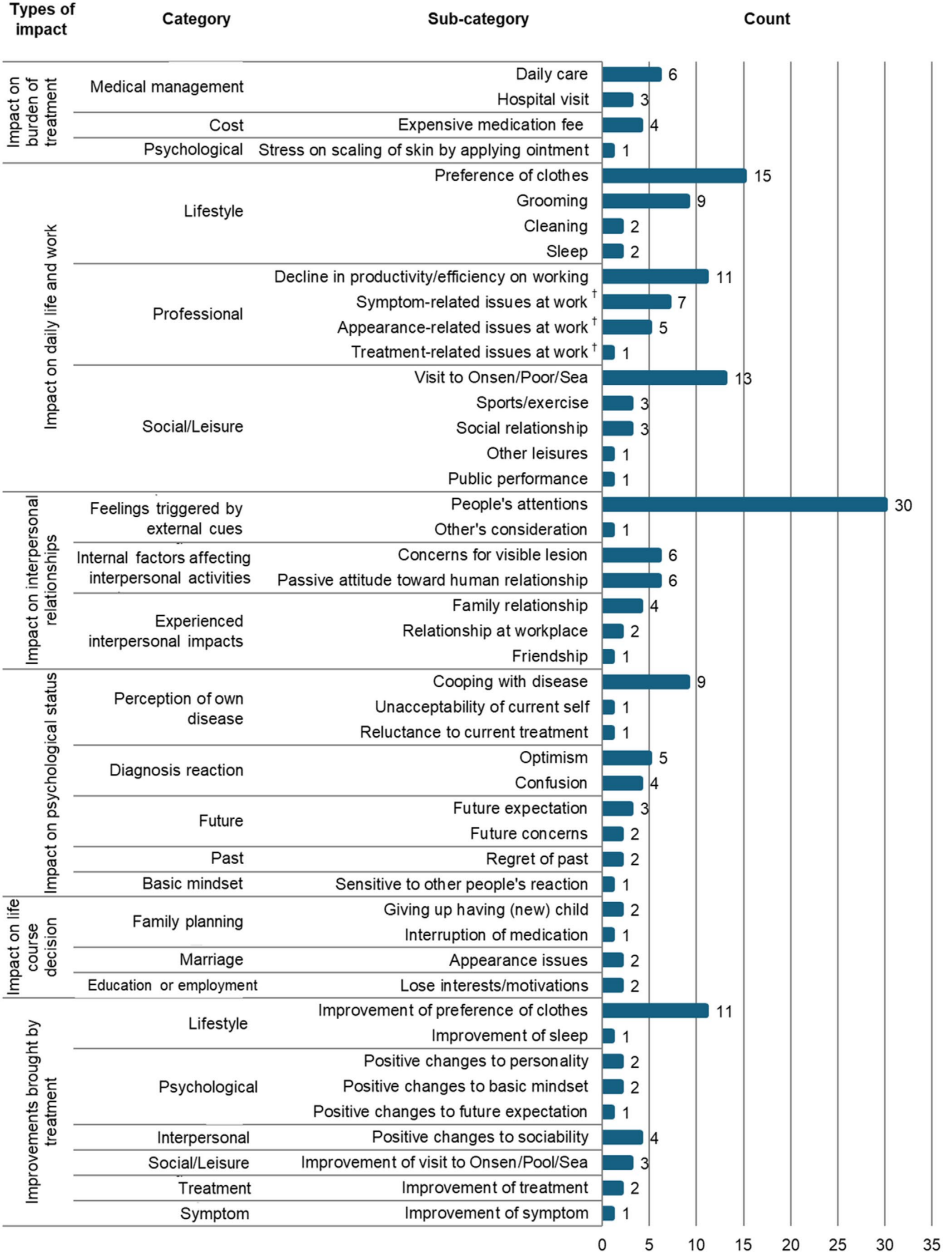
Frequency of sub‐categories, categories, and types of impact identified in 23 interviews. ^†^Other than work productivity/efficiency.

Six large concepts emerged: “burden of treatment,” “impact on daily life and work,” “impact on interpersonal relationships,” “impact on psychological status,” “impact on life course decision,” and “improvements brought by treatment” (Figure 1 and Table 2). Representative quotes for each concept are shown in Table S1.

**TABLE 2:** Concepts of psoriasis impacts: insights from patient interviews.

	Category	Sub‐category	Concept	*n*
Burden of treatment	Medical management	Day caring	Time consuming for daily care	6
		Hospital visit	Bothersome to visit hospital	3
	Cost	Expensive medication fee	Expensive medication fee	4
	Psychologist	Stress on scaling of skin by applying ointment	Stress on scaling of skin by applying ointment	1
				14
Impact on daily life and work	Life‐style	Preference of clothes	Covering arms/legs	7
			Avoid wearing short pants	3
			Preference of color	3
			Preference for hypoallergenic material/fabric	2
		Grooming	Consideration for hair care	5
			Ointment stains on clothes	3
			Started wearing makeup	1
		Cleaning	Frequency of cleaning up	2
		Sleep	Difficulty in sleep	2
	Professional	Decline in productivity/efficiency on working	Interruption due to symptom	5
			Interruption due to care/treatment	2
			Caring about the scaling of skin	2
			Interruption due to side effect	1
			Hardship in body temperature control	1
		Symptom‐related issues at work (other than work productivity/efficiency)	Negative impact on job performance	4
			Concerned about the sanitary condition of area around their desk	3
		Appearance‐related issues at work (other than work productivity/efficiency)	Ashamed/awkward of exposing skin lesions in visible body parts in workplace/in public space	5
		Treatment‐related issues at work (other than work productivity/efficiency)	Lack of understandings from colleague	1
	Social/Leisure	Visit to Onsen/Pool/Sea	Avoid going to Onsen/Pool/Sea	13
		Sports/exercise	Change in playing style	2
			Quit sports gym	1
		Social relationship	Avoidance of meet‐up or social activities	3
		Other leisure	Massage parlor	1
		Public performance	Avoidance of presentation in theatrical/stage performance	1
				73
Impact on interpersonal relationships	Feelings triggered by external cues	People's attentions	Concerned with misunderstanding from others	9
			Asked by people about the disease	8
			Concerned about people's perceptions	7
			Uncomfortable to attentions from others	6
		Other's consideration	Feel sorry for other's consideration	1
	Internal factors affecting interpersonal activities	Concerns for visible lesion	Hesitant showing the affected body part	4
			Embarrassed of lesion areas	2
		Passive attitude toward human relationship	Lack of motivation	4
			Avoidance from others	2
	Experienced interpersonal impacts	Family relationship	Feel sorry for inconvenience/additional burden due to disease	2
			Feel sorry for bad appearance	1
			Unable to fulfill family needs	1
		Relationship at workplace	Conflict due to misunderstanding of disease	1
			Being distanced by misunderstandings about the disease	1
		Friendship	Became estranged from friends	1
				50
Impact on psychological status	Perception of own disease	Coping with disease	Accept own disease	8
			Coping behavior with lesion areas	1
		Unacceptability of current self	Shocked for body‐image change	1
		Reluctance to current treatment	Stress on “difficult‐to‐treat”	1
	Diagnosis reaction	Optimism	Optimism for future	4
			Relief of having a diagnosis	1
		Confusion	Shocked by diagnosis	3
			Unfamiliarity to disease	1
	Future	Future expectation	Willingness to continue treatment	3
		Future concerns	Fear of future	2
	Past	Regret of past	Interruption of treatment	1
			Delay of switching treatment	1
	Basic mindset	Sensitive to other people's reaction	Stressed by attentions from others	1
				28
Impact on life course decision	Family planning	Giving up having (new) child	Giving up for financial reasons	1
			Concern of cumulative drug effect to the fetus	1
		Interruption of medication	Interruption of teratogenic medicine	1
	Marriage	Appearance issues	Appearance issues	2
	Education or employment	Lose interests/motivations	Lose interests/motivations for further education/employment	2
				7
Improvements brought by treatment	Lifestyle	Improvement of preference of clothes	Able to wear half sleeve shirts	6
			Able to wear dark clothes	3
			Able to wear white clothes	1
			Not concerned about scaling of skin	1
		Improvement of sleep	Better sleep with no ointment	1
	Psychological	Positive changes to personality	Be extrovert	1
			Be positive	1
		Positive changes to basic mindset	Less attention from people	2
		Positive changes to future expectation	Motivated for higher (treatment) goal	1
	Interpersonal	Positive changes to sociability	Lower barrier for meetup/going out	3
			Better appearance with no ointment	1
	Social/Leisure	Improvement of visit to Onsen/Pool/Sea	Able to go to Onsen/Pool/Sea	3
	Treatment	Improvement of treatment	Less burden of daily care	2
	Symptom	Improvement of symptom	Feel free from symptom	1
				27

Abbreviation: *n*, number of reports.

### 3.1 Burden of Treatment

The most reported treatment burden was “Day caring” (*n* = 6), specifically time allocation for ointment application. The financial burden (“Cost”) was also a concern (*n* = 4) with patients struggling to balance expenses and treatment efficacy.

### 3.2 Impact on Daily Life and Work

The most reported daily life impact was “Preference of clothes” (*n* = 14), with patients favoring garments that cover lesions, especially in summer. Many patients (*n* = 13) avoid public areas like onsen (public baths) or pools due to fears of attention or being perceived as infectious (*n* = 9). However, patients achieving treatment effects reported positive lifestyle changes, including both concepts. On work performance, 60% of employed patients reported impacts, including reduced productivity (*n* = 10) and interpersonal concerns like shedding skin fragments in the workplace.

### 3.3 Impact on Interpersonal Relationships and Psychological Status

Visible lesions increased attention from others, leading to “uncomfortable to attentions from others” (*n* = 6) and concerns about being misunderstood as having an infectious disease (*n* = 9). Patients frequently had to explain their condition (*n* = 8), adding to their social burden. Psychological reactions to diagnosis included optimism (*n* = 3) or shock (*n* = 3). Later on, patients were able to adjust to the disease/treatment after accepting that PsO is chronic or/and treatment is effective enough to manage daily life.

### 3.4 Impact on Life Course Decision

Three patients reported that PsO influenced decisions about family planning, including avoiding bearing children due to physical or psychological burdens. Others mentioned reduced motivation for life events due to visible symptoms.

## 4 Discussion

This qualitative descriptive study revealed the burden of visible PsO lesions and extracted meaningful PROs. Prior studies report impaired QoL in PsO patients, consistent with our findings [10, 11, 12, 13, 14]. PsO affects daily life, work, psychological aspects, relationships, and life decisions. Visible lesions influenced clothing choices, leisure activities, relationships, and self‐esteem, emphasizing the importance of prioritizing treatments that target visible symptoms. Expanding treatment options, such as oral and injectable medications that are easier and less time‐consuming than topical therapies, could improve adherence and satisfaction. However, balancing affordable price setting is crucial for continuing treatment sustainably, as cost is a major patient burden regardless of severity.

Work productivity was notably reduced, with 60% of employed patients reporting workplace challenges, including operational and interpersonal problems caused by visible symptoms like desquamation and blood stains on clothes. These findings suggest the need for understanding from coworkers.

Psychological impacts ranged from initial reactions of shock to long‐term adaptation. Due to chronic disease, patients go through a long journey. Understanding the psychological dynamics of the patients is crucial for healthcare providers to provide tailored support to address patients' emotional needs periodically.

A recurring concept was the stigma attached to visible skin lesions. Our findings also reflect the insufficient attention given to patients with PsO in Japan, pointing to a notable deficiency in disseminating medical knowledge about the condition. Addressing this problem requires awareness activities for PsO in schools and workplaces to the general public, alongside patient education could reduce stigma and support patients.

This study also highlighted that PsO influences life trajectories, including family planning, careers, and education, supported several studies that found that visible skin lesions and psoriatic joint lesions are more important for young patients when seeking employment or finding a long‐term partner [15]. Although the report of the influence of the life course decision was small, our results suggested the need to control/eliminate symptoms through early/proper treatment because disease onset in the teenage years may affect interest and vitality in schoolwork and employment.

The results of this study indicate that Japanese patients prioritize different aspects of treatment, highlighting the importance of confirming these needs. While assessing multifactorial disease burden in routine practice can be challenging, short, validated patient‐reported outcome (PRO) tools, such as DLQI and VAS for key symptoms, may provide a practical solution. In addition, digital health technologies such as mobile applications and patient portals may facilitate PRO data collection outside of clinic and allow patients to report their experiences conveniently. However, unique findings in this study, such as concerns over being misunderstood, highlight the need for more comprehensive tools. A questionnaire more suited to the Japanese population needs to be developed to assess the burden comprehensively and effectively.

Limitations include recall bias, with 65% of patients diagnosed over 10 years ago, and a predominantly older sample, which limits insights into life events for younger patients. This study focused on cases with visible skin lesions, but without control subjects, it is difficult to confirm whether the findings are specific to such cases. A small sample size also restricts generalizability. Future studies should expand the sample size and incorporate quantitative methods using validated scales.

The multifaceted impact of PsO in Japan emphasizes the need for holistic care that addresses practical, psychological, and social challenges. Tailored interventions and public awareness efforts are crucial for reducing stigma and misconceptions. Further study is needed to deepen the understanding of the unique burden of PsO to improve the care and QoL of Japanese patients.

## Ethics Statement

This study was conducted according to the Ethics Committee of the Non‐Profit Organization Tokyo Allergy and Respiratory Disease Research Institute, with approval number 20230701. Before the interview was administered, informed consent was obtained from all participants. This study is registered with UMIN‐CTR under the registration number UMIN000051977. No animal studies were conducted in this research.

## Conflicts of Interest

Author K.E. has received personal fees from AbbVie, Kyowa Kirin, Sanofi, Sun Pharma, Celgene, Taiho Pharmaceutical, Novartis, Torii Pharmaceutical, Otsuka Pharmaceutical, Eli Lilly, Pfizer, Bristol Myers Squibb, Maruho, UCB, LEO Pharma, and Janssen. Author Y.H. has conducted the study with AbbVie. Author A.C. is a former employee of AbbVie and may own stock or stock options in the company and is now an employee of RICOH CREATIVE SERVICE COMPANY LTD. Author Y.S. and K.I. are employees of AbbVie G.K. Author M.O. and M.M. are employees of Syneos Health Clinical, a contract research organization with clients in the biotech/pharmaceutical industry. Author Y.T. has received research fees and an honorarium from Kyowa Kirin, Eli Lilly Japan Co. Ltd., AbbVie, Maruho, Amgen, Taiho Pharmaceutical Co. Ltd., Mitsubishi Tanabe Pharma, Novartis, Sanofi, UCB Japan Co. Ltd., Torii Pharmaceutical Co. Ltd., Leo Pharma Co. Ltd., Eisai Co. Ltd., Takeda Pharmaceutical Co. Ltd., Nippon Boehringer Ingelheim Co. Ltd., JIMRO, Bristol Myers Squibb Co. Ltd., Sun Pharma Co. Ltd., and Tokiwa Pharmaceutical Co. Ltd., research fees from Smith & Nephu Co. Ltd., Japan Blood Products Organization, Mochida Healthcare Co., and Sato Pharmaceutical Co. Ltd. and an honorarium from Janssen Pharmaceutical. Also, author Y.T. is an Editorial Board member of The Journal of Dermatology and a co‐author of this article. To minimize bias, she was excluded from all editorial decision‐making related to the acceptance of this article for publication.

## Supporting information


Table S1.
